# Effect of One Year COVID-19 on Trauma of Lower Extremity at Orthopaedic Service in Prof Soeharso Orthopaedic Hospital, Indonesia: A Cross-sectional Study

**DOI:** 10.5704/MOJ.2211.003

**Published:** 2022-11

**Authors:** P Utomo, A Santoso, AG Faza, MB Yudhistira

**Affiliations:** Faculty of Medicine, Universitas Sebelas Maret, Surakarta, Indonesia

**Keywords:** COVID-19, trauma of lower extremity, orthopaedic hospital

## Abstract

**Introduction:**

The World Health Organization announced the COVID-19 outbreak as a global pandemic on March 11, 2020. Despite the fact that orthopaedic departments are not considered first-line department in the war against pandemic, the pandemic has had a big effect on orthopaedic services. A few studies have found the pandemic effect on the orthopaedics field, but none have found the effect of a one-year pandemic, especially in Indonesia. This study aimed to know the effect of one-year COVID-19 on trauma of lower extremity at Orthopaedic Service in Prof Soeharso Top Referral Orthopaedic Hospital, Indonesia

**Materials and methods:**

It is a cross-sectional study. The study compared the population group during one year of the COVID-19 pandemic in Indonesia to the same period one year before. This study was conducted in Prof. Dr. R. Soeharso Orthopaedic Hospital, Surakarta, Indonesia from March 2019-February 2021. The subjects were patients of lower extremity trauma both surgical procedure and outpatient visit. Patients recorded on other orthopaedic service support installations like radiology, laboratory, or physiotherapy were excluded.

**Results:**

There was a significant reduction (54.9%) in total trauma of lower extremities patients in Prof Dr R Soeharso Orthopaedic Hospital, Surakarta, from 2146 (pre-COVID-19) to 968 (during COVID-19) in the March 2019-February 2021 period. There was also a significant reduction (90.9%) in total cases outpatient visit in pre-COVID-19 compared to during COVID-19 (p<0.05) and surgical procedures (39%) in pre-COVID-19 compare to the COVID-19 period (p<0.05).

**Conclusion:**

There was a significant reduction on trauma of lower extremities patients both outpatient visits and surgical procedures during pandemic COVID-19 than before the COVID-19 occurred.

## Introduction

Coronavirus disease 2019 (COVID-19) is a disease caused by the transmission of Severe Acute Respiratory Syndrome Coronavirus 2 (SARS-CoV-2)^1^. COVID-19 is a respiratory disease that causes symptoms like common cold, fever, dry cough, shortness of breath, and in extreme cases, SARS. Productive cough and gastrointestinal problems are two less common signs^2^. Moreover, COVID-19 reduced the number of services in orthopaedic and trauma departments^3^. Likewise, Hongkong and Ireland studies indicated decreased number of trauma and orthopaedic service during the COVID-19 pandemic^4^. However, no one has investigated the effects of the COVID-19 pandemic on the orthopaedic services after a year in Indonesia.

Therefore, this study aimed to analysing how COVID-19 affects orthopaedic services in lower extremity trauma at Prof. DR. R. Soeharso Orthopaedic Hospital Surakarta.

## Materials and Methods

This research is a cross-sectional comparative analysis study. The study compared the population group during one year of the COVID-19 pandemic (March 2020 - February 2021) with the same period one year before (March 2019 - February 2020). This study took place in National Referral Orthopaedic Hospital, Prof. Dr. R. Soeharso Orthopaedic Hospital, Surakarta, Indonesia. The study was carried out for three months, from February 2021 until April 2021.

Data in this study was primary data, collected from hospital digital medical records. First, anonymised records were gathered for one year from the first month's Indonesian government’s declaration of the first COVID-19 infection case on its people (February 2020 – February 2021). Then, the data from one year before the COVID-19 pandemic were served as the control (March 2019-February 2020). Predefined characteristics: dates, primary and secondary diagnosis, surgical therapy, aetiology, supervisor, and installation were extracted. Besides, the nomenclature for diagnosis and therapy was based on ICD-10.

The primary outcomes of this study were the number of patients that require orthopaedic service on trauma of lower extremity. The inclusion criteria of this study were all patients who came to Prof. Dr. R. Soeharso Orthopaedic Hospital with a trauma of lower extremity-related diagnosis from the emergency department, outpatient visit, inpatient and the operating room. However, patients recorded on other orthopaedic service support installations like radiology, laboratory, or physiotherapy were excluded.

Following ethics approval, the data were gathered from the medical records of Prof. Dr. R. Soeharso Orthopaedic Hospital based on inclusion and exclusion criteria. The collected data were then classified based on the predefined characteristic aforementioned. Statistical analysis was employed to compare the data. In this case, different conditions before and during pandemics were compared. The difference between multiple time points (Months) during the pandemic was also calculated.

Statistical data were analysed utilising an independent sample t-test or Mann-Whitney. Analysis was undergone in Statistical Package for the Social Sciences (SPSS) 26.0 program for Windows manufactured by IBM in New York, USA. The difference between the number of outpatient visits and surgical procedures between the two groups was considered statistically significant if p<0.05.

## Results

**Fig. 1.**
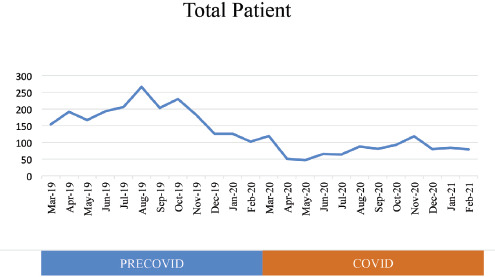
Total Trauma of lower extremities patients of the Prof. Dr. R. Soeharso orthopaedic hospital.

A total of 3114 patients was enrolled in this study. The mean of lower extremity trauma patients during the pre-COVID-19 period was 89.42 (Standard Deviation [SD] 44.68) patients, and during the COVID-19 period was 40.33 (SD 39.12) patients. Total patients of trauma of lower extremities both pre-COVID-19 and COVID-19 period will be shown in (Fig. 1) and its statistic in (Table I).

**Table I:** Total lower extremity patient pre-COVID-19 and COVID-19 period

	Pre-COVID-19	Mean COVID-19	*p*-values*
Total Cases of Lower Extremity (Outpatient and Surgery Procedure) (SD)	89.42 (44.68)	40.33 (39.12)	<.001

*Independent sample t-test

There was a significant reduction (54.9%) in the total patients of lower extremity trauma from 2146 in the pre-COVID-19 period (March 2019-February 2020) to 968 in the COVID-19 period (March 2020 - February 2021) (p<0.05). Likewise, total outpatients' visits and surgical procedures in lower extremity trauma decreased drastically compared to the same period in 2019 (p<0.005).

**Fig. 2.**
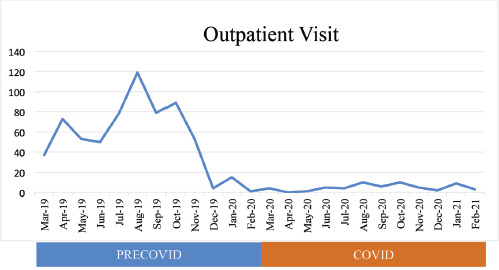
Total outpatient visit in the lower extremity section of the Prof. Dr. R. Soeharso orthopaedic hospital.

**Fig. 3.**
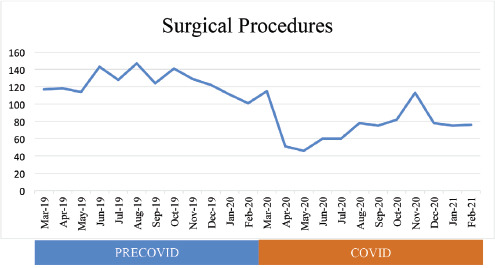
Total surgical procedures in the lower extremity section of the Prof. Dr. R. Soeharso orthopaedic hospital.

The total outpatient visits significantly reduced (90.9%) from 651 in the pre-COVID-19 period up to 59 in the COVID-19 period (p<0.05). The month of April 2020 had the lowest number of outpatient visits, with zero total visit. The total surgical procedures significantly reduced (39%) from 1495 in the pre-COVID-19 period up to 909 in the COVID-19 period (p<0.05). The month of May 2020 had the lowest number of surgical procedures, with 46 total visits. The data will be shown in (Fig. 2 and 3) and its statistic, it will be shown in (Table II).

**Table II:** Total outpatient visit and surgery procedure in pre-COVID-19 and COVID-19 period

Classification		Pre-COVID-19	Mean COVID-19	*p*-values*
Outpatient Visit	Total Cases (SD)	54.25 (35.86)	4.92 (3.34)	<.001
Surgery	Total Cases (SD)	124.58 (13.82)	75.75 (21.26)	<.001

*Independent sample t-test

Further, based on the region of these outpatient and surgical procedures in lower extremity trauma, four subsections were defined: thigh, knee, lower leg, and foot. There was a significant reduction in cases of lower extremity trauma based on the region of thigh, knee, lower leg, and foot, both surgical procedures and outpatient visits (p<0.05), their data will be shown in (Table III and IV).

**Table III:** Total surgical procedure patients in pre-COVID-19 and COVID-19 period by region

Surgical Procedures		Pre-COVID-19	Mean COVID-19	*p*-values
Regio	Thigh (SD)	38.08(5.69)	22.75(5.49)	<.001*
	Knee (SD)	19.83(2.69)	12.00(5.98)	<.001*
	Lower Leg (IQR)	19.83 (4)	12.00 (9)	.713**
	Foot (IQR)	3.92 (5)	2.08 (2)	.005**

*Independent sample t-test

**Mann Whitney

**Table IV:** Total outpatient visits in pre-COVID-19 and COVID-19 period by region

Outpatient Procedures		Pre-COVID-19	Mean COVID-19	*p*-values
Regio	Thigh (SD)	29.00 (19.42)	2.33 (1.49)	<.001
	Knee (SD)	16.92 (13.29)	1.42 (2.10)	.002
	Lower Leg (IQR)	3.42(2.74)	.42(.79)	.003
	Foot (IQR)	4.58 (4.92)	.75 (1.05)	.022

*Independent sample t-test

## Discussion

This research’s findings showed that total patients decreased significantly in each month during COVID-19 compared to total patients during the comparison period (p<0.05). In addition, this study’s data revealed that the number of outpatients visit of lower extremity trauma cases decreased significantly in April 2020, with zero case reported and 46 cases reported in surgical procedures in May 2020. These results might happen because of large-scale social restrictions including mobility restriction, stay at home, and work from home^5^. These findings support the previous research that found a significant decline in inpatient visits in Austria since the pandemic^6^. Another report backs up this research’s findings, stating that the number of visits to the emergency department in Hong Kong decreased significantly from the same time on the previous year, including emergency and life-threatening cases like acute coronary syndrome^7^. Supporting this current study, a study showed that a total of 405 patients were admitted to the hospital over three months in both periods (pre-COVID-19: 269 patients and COVID-19: 136 patients), indicating that lockdown made reduced visits by 50% in the COVID-19 period. Then, the number of surgical procedures during the COVID-19 period (n=129) was 50% less than the pre-COVID-19 period group (n=261). Surgical procedures related to trauma also reduced from 133 to 81 from the pre-COVID-19 period to the COVID-19 period. More details, lower limb surgical procedures during COVID-19 era (n=27) were 49% less compared to pre-COVID-19 period group (n=53)^8^. When the Indonesian limitation activity policy, known as Pembatasan Sosial Berskala Besar (PSBB), began on 31 March 2020, and a month later, the total number of patients dropped dramatically. In contrast, a study from the United Kingdom showed no changes in the number of trauma injuries that needed plastic surgery procedures during the lockdown period^9^.

At the first time WHO declared a COVID-19 pandemic on March 11, 2020, with more than 118 000 cases spread across 114 countries and 4291 deaths, Indonesia has implemented various policies^10^. In this case, various sectors get the impact of these policies. One of them is in the orthopaedic service in lower extremity trauma cases comparing between pre-COVID-19 and during the COVID-19 period. After Mr. Joko Widodo, the president of the Indonesian government, enforced large-scale social restrictions on March 31, 2020, including mobility restriction, stay at home, and work from home, the number of patients decreased in April-May 2020 compared to the previous month in the pre-COVID-19 period^5^. The reduction in lower extremity patient trauma might happen because of various factors, including government, hospital, and community efforts. In the hospital sector, programs such as suspending operations for prioritising patient safety and surgery over elective surgery were carried out to reduce the risk of COVID-19 transmission^11^. In line with that, in the COVID-19 pandemic, people with mild conditions were discouraged from going to healthcare facilities to reduce transmission risk. Likewise, since prolonged hospitalisation raises the risk of COVID-19 infection, some hospitals advised patients to delay or cancel elective surgery^12^. In the government policy sector, large-scale social restrictions, such as closing schools and workplaces, prohibiting physical worship, stay at home, and limiting public meetings, were done to reduce the number of people affected by COVID-195. In the community sector, because of the COVID-19 pandemic, most people stayed at home. The patient might be fearful of contracting COVID-19 and avoid going to the hospital^11^. Likewise, people feared contracting the disease in the potentially crowded emergency department in the hospital^13^. Thus, it reduced the number of outpatient visits and surgical procedures in hospitals^14^. The hospital, government policy, and community sectors have a role in reducing the number of hospital outpatient visits and surgical procedures.

Within this current study, the reduction of cases happened in both surgical procedures and outpatient visits of lower extremity by region (femoral, genu, cruris, and metatarsal). Supporting this current study, a study showed the number of hospital visits and surgical operations for upper and lower limb fractures decreased, with decreases seen in all types of traumas, including sports, road, manufacturing, and domestic accidents^15^. Also, there was a significant reduction (p<0.05) of cases in lower limb trauma by region (proximal femur fractures, femur and tibia shaft fractures, foot and ankle fractures, distal femur and proximal tibia fractures) in COVID-19 period compared to pre-COVID-199. The region of injury and number of cases have all changed because of COVID-199.

As this study was held at the top national referral hospital of orthopaedic services in Indonesia, it is expected to represent quite relevant data at the national level. The researchers believe that this study may contribute to healthcare policies in the future pandemics.

Considering the limitation of this study, it is necessary to consider the differences in activity limitation policies in each country. The studies mentioned above were conducted in countries with stricter quarantine or lockdown policies than Indonesia. Because of the effectiveness of each policy, the outcomes may differ. The habit of the people using traditional medicine also needs to be considered a factor influencing this study's findings.

## Conclusion

There was a significant reduction on trauma of lower extremities patients both outpatient visits and surgical procedures during pandemic COVID-19 than before the COVID-19 occurred. Regular training for all hospitals about pandemic readiness is necessary in the future to improve service optimality and further research is needed to identify the efficacy of PSBB compared to other nation’s activity limitation policies.
